# Assessment of Hair Aluminum, Lead, and Mercury in a Sample of Autistic Egyptian Children: Environmental Risk Factors of Heavy Metals in Autism

**DOI:** 10.1155/2015/545674

**Published:** 2015-10-05

**Authors:** Farida El Baz Mohamed, Eman Ahmed Zaky, Adel Bassuoni El-Sayed, Reham Mohammed Elhossieny, Sally Soliman Zahra, Waleed Salah Eldin, Walaa Yousef Youssef, Rania Abdelmgeed Khaled, Azza Mohamed Youssef

**Affiliations:** ^1^Pediatrics Department, Faculty of Medicine, Ain Shams University, Cairo, Egypt; ^2^National Institute of Standards, Giza, Egypt; ^3^Community Medicine Department, Ain Shams University, Cairo, Egypt

## Abstract

*Background and Aims*. The etiological factors involved in the etiology of autism remain elusive and controversial, but both genetic and environmental factors have been implicated. The aim of this study was to assess the levels and possible environmental risk factors and sources of exposure to mercury, lead, and aluminum in children with autism spectrum disorder (ASD) as compared to their matched controls. *Methods*. One hundred ASD children were studied in comparison to 100 controls. All participants were subjected to clinical evaluation and measurement of mercury, lead, and aluminum through hair analysis which reflects past exposure. *Results*. The mean Levels of mercury, lead, and aluminum in hair of the autistic patients were significantly higher than controls. Mercury, lead, and aluminum levels were positively correlated with maternal fish consumptions, living nearby gasoline stations, and the usage of aluminum pans, respectively. *Conclusion*. Levels of mercury, lead, and aluminum in the hair of autistic children are higher than controls. Environmental exposure to these toxic heavy metals, at key times in development, may play a causal role in autism.

## 1. Introduction

The* autism spectrum* describes a range of conditions classified as neurodevelopmental disorders in the fifth revision of the American Psychiatric Association's* Diagnostic and Statistical Manual of Mental Disorders 5th Edition* (DSM-5). These disorders are characterized by social deficits and communication difficulties, stereotyped or repetitive behaviors and interests, sensory issues, and in some cases cognitive delays [1].

The increase of ASDs prevalence cannot be fully explained by advances in diagnostics or sudden genetic shifts. There is a growing consensus among scientists and clinicians that ASDs ensue from an interaction between biological vulnerability factors and environmental or iatrogenic insults [2].

This points to the importance of environmental factors and raises the possibility of an etiological role for toxic exposures: either prenatal, postnatal, or in some cumulative pattern that combines the effect of maternal, gestational, and infant exposures [3].

Some possible sources of heavy metal poisoning include chemical products, fertilizers, industrial paint, building materials, fish that is high in mercury, silver dental fillings, and mercury-containing preservatives (thiomersal) in vaccines. Lead may be found in the dirt near roads and can still be found in paint from older houses. Children eating paint chips or those with pica may develop toxic lead levels [4].

Genetically, children with autism may be less able to detoxify toxic environmental agents, and this inability may predispose them to suffer neural damage consistent with autistic behavioral traits [4].

Women with chronic metal exposure (who have accumulated high tissue levels of mercury and other metals) may pass potentially toxic metals to their fetuses or intoxicate infants through nursing [5].

We conducted the study to examine the possible risk factors and sources of exposure to mercury, lead, and aluminum in children with autistic spectrum disorder and assess the levels of heavy metals in hair of both autistic and control groups.

## 2. Participants

This case control study included one hundred autistic children (84 boys and 16 girls); their ages ranged from 2.5 to 15 years with mean of 6.2 ± 2.4 years. The children were diagnosed according to the DSM-IV TR (2000) criteria by pediatric psychiatrists in the Child Psychiatry Clinic, Children's Hospital, Ain Shams University from December 2011 to December 2014. A control group was selected, which included one hundred age- and gender-matched healthy children. These children were friends and neighbors, unrelated to the study group.

A written consent from the parents and bioethical research committee approval were taken. Patients were excluded from the study if they were suffering from liver or kidney disease, anemia, or current treatment for iron deficiency, progressive neurological disorders, or unstable epilepsy. Also children with mercury dental amalgam, previous use of DMSA or other chelators were excluded.

All of the children admitted to the study received routine childhood vaccinations.

## 3. Methods

All children in the current study were subjected to the following:* detailed history* taking with special emphasis on antenatal or maternal history asking about maternal dietary habits (the type and amount of fish consumption by the mother during pregnancy especially canned tuna fish and the imported frozen mackerel fish (the cheapest fish in Egypt)), maternal dental work (filling amalgam or removal), and if Rho(D) immune globulin was given during pregnancy.

Developmental history was taken laying stress on all developmental milestones (gross motor, fine motor, sphincter control, language, cognitive development, and social milestones). Also, behavioral disorders (history of pica, stereotypic behavior) and dietary history (being breast fed or artificially fed, duration, weaning history, and problems during weaning) were noted.

Potential environmental toxic exposures were particularly noted such as gasoline station in close proximity to the child's home, cooking habits (type of utensils used especially aluminum pans), and the age of the patients' house (type of paint and water pipes).

Also, past history of major childhood illnesses and immunizations was taken.

Thorough* clinical examination* of all body systems with special emphasis on neurological examination.

All autistic children were subjected to a full* clinical child psychiatric evaluation* for diagnosis of autistic spectrum disorder and exclusion of other psychiatric disorders according to* Diagnostic and Statistical Manual of Mental Disorders 4th Edition*,* Text Revision* (DSM-IV-TR) [6].

The severity of autistic symptomatology was measured by the Childhood Autism Rating Scale (CARS) [7]. It consists of 15 categories, each rated on a four-point scale. The individual is considered nonautistic when his total score falls in the range of 15–29, mildly-to-moderately autistic when his total score falls in the range of 30–36, and severely autistic when his total score falls in the range of 37–60 [8]. Based on the administration of multiple assessments, insights into various aspects of autism are gained.

IQ assessment (intelligent quotient) using* Stanford-Binet Intelligence Scale* [9]: ranges of IQ are as follows: 20–30 denotes severe mental retardation, 31–49 is moderate mental retardation, 50–70 is mild mental retardation, 71–89 is below average, 90–109 is normal IQ, and 110–125 is above average.

*Hair Specimen Collection*. We decided to use hair mineral analysis to evaluate the long term metal exposure. Hair sampling is a noninvasive technique; it is the best indicator of a given mineral in the body. These samples were collected from cases and control by single cutting from the occipital region. The samples were cut to lengths of about 1.5–2 cm using clean stainless steel scissors. A minimum of 5–10 mg of hair was required for the hair analysis assay. Approximately 100 strands of hair (50 mg) were used. Adhesive paper was placed over the end of the hair strands closest to the scalp; the paper was marked with an arrow indicating the end of hair closest to the scalp. The samples were placed in a sealed plastic bag [10].

*Instrumentation*. The measurements of lead and aluminum were made by Electrothermal Atomic Absorption Spectrometer Zeenit 700 (Germany) equipped with Zeeman background correction and automatic autosampler. Hydride generation technique was used for mercury measurements.

### 3.1. Materials and Reagents

Calibration solutions were prepared from Pb, Hg, and Al certified reference materials of concentration 1000 mg/L maintained at National Institute of Standards (NIS):

- concentrated nitric acid with purity of 69%,
- sodium borohydride with purity 98%,
- sodium hydroxide with purity of 99%,
- ammonium phosphate monobasic 1% which is prepared to be used as a modifier for lead measurements by electrothermal atomic absorption spectrometer (AAS).

#### 3.1.1. Sample Preparation

Hair samples were washed with pure acetone and three times with ultrapure water and put in a drying furnace at 70°C over night. After cooling, the samples were caught in small pieces of 2 mm length. About 0.15–0.20 g of hair samples was weighed, mixed with 2 mL concentrated nitric acid, and left in a furnace at 90°C for 24 hours. After cooling, the samples were transferred to 25 mL measuring flask and completed by ultrapure water. Further dilution was required when the concentrated range exceeded the calibration range. Test values were reported in mg/kg.

#### 3.1.2. Statistical Methodology

The collected data was revised, coded, tabulated, and introduced to Statistical Package for Social Science* (SPSS 15.0.1 for windows; SPSS Inc., Chicago, IL, 2001)*. Data was presented and suitable analysis was done according to the type of data obtained for each parameter:

(1) * Descriptive statistics:* mean standard deviation (±SD) and range were used for parametric numerical data, while median was used for nonparametric numerical data and frequency and percentage of nonnumerical data.
(2) * Analytical statistics:* Student's* t*-test was used to assess the statistical significance of the difference between two study group means,* Mann-Whitney test (U test)* was used to assess the statistical significance of the difference of a* nonparametric* variable between two study groups,* ANOVA test* was used to assess the statistical significance of the difference between more than two study group means,* the Kruskal-Wallis* test is was used to assess the statistical significance of the difference between more than two study group* nonparametric* variables,* Chi-square test* was used to examine the relationship between two qualitative variables,* and Fisher's exact test* was used to examine the relationship between two qualitative variables when the expected count is less than 5 in more than 20% of cells.
(i) *P* value of 0.05 was considered significant.

## 4. Results

Table 1 shows that the autistic group consisted of 84 boys (84%) and 16 (16%) girls; their mean age was 6.24 ± 2.4 years. The control group consisted of 74 (74%) boys and 26 (26%) girls with the mean age of 6.8 ± 3.04 years.

There were significant differences regarding the amount of sea food eaten per month by mothers during pregnancy (*P* = 0.0001). Also, there were significant differences regarding exposure to other environmental risks in pregnancy as maternal exposure to immunoglobulin D (anti-D), dental amalgam, painting, old house age, and maternal smoking (*P* = 0.002, 0.001, 0.001, and 0.0001, resp.). But there were no significant differences in infancy regarding vaccination (*P* = 1), Table 1.

Table 2 shows a significant increase in the maternal age of autistic group versus the controls (*P* = 0.001). Also there were statistically significant differences in the mean duration of breast feeding as autistic patients had less duration of breast feedings than controls (*P* = 0.001), Table 2.

**Figure 1:**
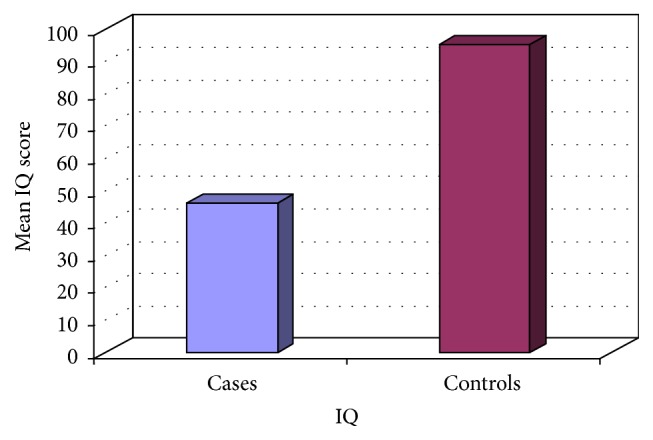
Comparison between cases and controls as regards IQ score, *P* = 0.0001, Student's* t*-test.

Figure 1 shows that autistic children had lower IQ scores than the controls (*P* = 0.0001).

**Figure 2:**
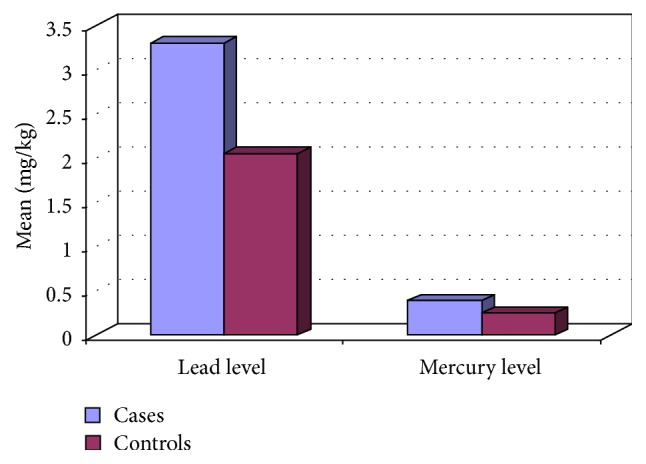
Mean lead and mercury levels in hair in both groups.

**Figure 3:**
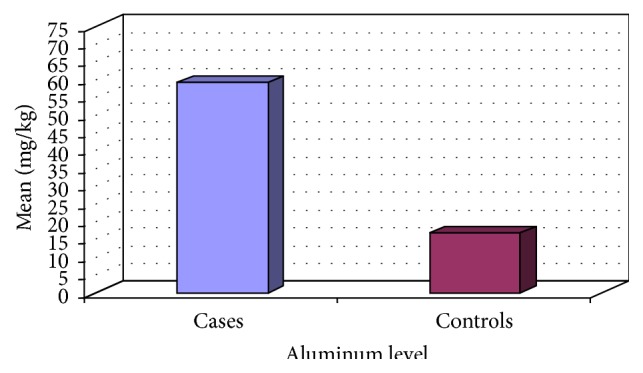
Mean aluminum level in hair in both groups.

Comparison between cases and controls as regards heavy metal levels shows that the mean levels of the three toxic heavy metals in hair were significantly higher among the studied ASD cases than the controls (*P* = 0.015, 0.023, and 0.0001 for mean levels of mercury, lead, and aluminum, resp.), Table 3 and Figures 2 and 3.

On examining the relationship between environmental risk factors and toxic heavy metals levels among cases we found the following: there was a significant relation between fish consumption in the patient and the mercury levels; also the nearby gasoline stations affected the lead levels, while aluminum pans usage increased aluminum level in the studied autistic patients (*P* = 0.026, 0.047, and 0.04, resp.), Tables 4, 5, and 6.

An important finding in the current study was the absence of significant association between the severity of the autistic manifestations, as measured by the CARS scale, in cases and the heavy metals level, Table 7.

## 5. Discussion

The current study was conducted on 100 autistic children; their ages ranged from 2.5 to 15 years with a mean 6.4 ± 2.4. Eighty-four percent of them were males and 16% were females, with a male/female ratio of 4 : 1.

On comparing between the levels of lead, mercury, and aluminum in hair of the autistic patients and the controls, the levels were significantly higher among cases than controls (*P* < 0.015, 0.023, and 0.0001, resp.).

In agreement with these results were Fido and Al-Saad [11] in Kuwait, Al-Ayadhi [12] in Riyadh, and El sheshtawy et al. [13]* in Egypt*.

According to Geier et al. [14], 58 research articles provided empirical evidence relevant to the question of a link between an ASD diagnosis and one or more toxic metal exposures; 74% of the studies examined showed a significant relationship between an ASD diagnosis and toxic metal exposure. These investigators concluded that the balance of studies supports a link between ASD diagnoses and toxic metal exposure.

Contrary to these results some studies reported that there was no correlation between a higher body burden of toxic metals and an ASD diagnosis, Abdullah et al. [15] and Albizzati et al. [16]. Moreover, Holmes et al. [17] and Kern et al. [18] found that hair excretion patterns of heavy metals among autistic infants were significantly reduced relative to control.

*As regards Mercury (Hg).* In the current study, the mean mercury level of the autistic patients (0.39 ± 0.37 mg/kg) was significantly higher than that of the controls (0.25 ± 0.16 mg/kg) with *P* = 0.023; this was consistent with other studies assessing the increased mercury body burden/toxicity in subjects diagnosed with the autism spectrum [4, 14, 19, 20].

In the present study we correlated some risk factors associated with mercury toxicity and our autistic children. As regards maternal fish consumption during pregnancy, it was significantly higher than that of the controls (*P* = 0.0001). And there was a statistically significant increase in the hair mercury levels as fish consumption increased (*P* = 0.026). This is in agreement with the results of [21–23]. Moreover, Fang et al. [24] found that hair mercury concentration was positively associated with the average mass of fish consumed weekly, indicating that fish consumption is the main contributor to hair Hg.

The present study, also, showed that the maternal use of dental amalgam was statistically more than controls (*P* = 0.002). Although mercury levels increased as the maternal use of dental amalgam increased in autistic patients, this increase did not reach statistical significance (*P* = 0.477). Studies done by [4, 25] found that mercury from maternal amalgam fillings resulted in a significant increase of mercury concentration in the tissues and the hair of fetuses and newborn children. Furthermore, placental, fetal, and infant mercury body burden was correlated with the numbers of amalgam fillings of the mothers.

Contrary to our results [26–28] reported that there was no evidence that exposure to dental amalgam was associated with impaired neuropsychological functions.

Although there was a significant increment of mercury level in autistic cases with the maternal use of Rho(D) immune globulin, this increment was not statistically significant (*P* = 0.239). This finding was supported by [29] that revealed a significant association between total mercury exposure during the prenatal and early postnatal periods from thimerosal-containing immunoglobulins and the severity of autism. Also, studies done [21, 26] demonstrated the same results. On the other hand, [30, 31] found that prenatal exposure to thimerosal-containing Rho(D) immune globulin does not increase the risk of autism.

*As regards Lead (Pb).* In the current study, the mean lead level in the autistic patients (3.31 ± 3.92 mg/kg) was significantly higher than that of the controls (2.06 ± 2.45 mg/kg) with *P* = 0.015. Jiang et al. [32] studied heavy metal concentrations in hair of preschool autistic children and found that hair lead concentration was significantly elevated.

The current study showed a statistical increase in lead levels with a presence of nearby gasoline stations (*P* < 0.047). This is in agreement with this result, a study done by Naeher et al. [33] who demonstrated that the lead levels of children living nearby gas stations were marginally higher than for children living away from gas stations.

*As regards Aluminum (Al).* In the current study, the mean aluminum level in the autistic patients (59.19 ± 37.98 mg/Kg) was significantly higher than that of the controls (16.78 ± 17.3198 mg/Kg) with *P* = 0.0001. This is in agreement with Tomljenovic and Shaw [34], who showed that Al, a highly neurotoxic metal and the most commonly used vaccine adjuvant, may be a significant contributing factor to the rising prevalence of ASD in the Western world.

As regards the usage of aluminum pans, it was significantly higher in the studied cases than the controls (*P* = 0.005). Abu-Taweel et al. [35] documented experimentally that perinatal oral Al exposure including use of aluminum pans, particularly during pregnancy and lactation period, can affect the in utero developing fetus of mice. So aluminum exposure has potential and long lasting neurotoxic hazards and might modify the properties of the dopaminergic system and thus can change the threshold of that system or other related systems at later ages.

An interesting result in our study was the absence of statistically significant relation between the levels of mercury, lead, and aluminum and autism severity. This was not consistent with [13, 36] that found that, on comparing hair concentration of autistic cases versus controls, elevated hair concentrations were noted for heavy metals in autistic children and correlated with the severity of symptoms. Also, Adams et al. [37] found that the body burden of toxic metals was significantly related to the variations in the severity of autism. The metals of greatest influence were lead (Pb), mercury (Hg), and aluminum (Al). Geier et al. [38] suggested that the impact of toxic metals may be more evident in subjects diagnosed with moderate to severe ASD as opposed to participants diagnosed with a mild ASD.

It may be argued that children with ASD are not the only children exposed to potentially toxic metals; the reason why autistic patients show greater concentration of potentially toxic metals in tissue may be the result of a greater ability to accumulate toxins, which in turn leads to an alteration of biochemical processes. Also, children with autistic spectrum disorders displayed lower levels of the nutritional elements calcium, copper, chromium, manganese, magnesium, iron, selenium, and cobalt. Since autistic children display poor eating habits, the low tissue levels may be explained by an inadequate nutritional intake.

Therefore, it is believed that ASD patients have problems with the chemical pathway that allows them to detoxify metals to alleviate different cluster of autistic symptoms [39]. Evidence shows that autistic children show an increased build-up of toxins which may not arise simply from excessive exposure but from a marked inability to process and eliminate toxins from the body. Such a mechanism could lead to a back-up of toxic heavy metals and chemical toxins and increases free radical activity in the body [40].

Adams et al. [37] observed that toxic metal excretion pathways may significantly vary among study subjects diagnosed with moderate to severe ASD as opposed to participants diagnosed with a mild ASD. This may be of a particular importance when examining hair toxic metal concentrations in young children because previous studies have suggested that hair toxic concentrations may be related to toxic metal excretion rates. Emerging evidence supports the theory that some ASDs may result from a combination of genetic/biochemical susceptibility, specifically a reduced ability to excrete mercury (Hg), and exposure to Hg at critical developmental periods [4, 38, 41].

Exposure to Hg can cause immune, sensory, neurological, motor, and behavioral dysfunctions similar to traits defining/associated with ASDs, and these similarities extend to neuroanatomy, neurotransmitters, and biochemistry [4, 38, 41]. Pediatric lead poisoning has deleterious effects on the development of widespread brain areas including those implicated in cognitive, communication, and social functioning [42].

The brain is the organ most sensitive to lead exposure [33]. Lead poisoning interferes with the normal development of a child's brain and nervous system. Biological damage from toxic material and increased environmental exposure at key times in development may play a causal role in the etiology of autistic disorders and potentially increases the severity of autistic symptoms.

In 2009, Blaylock and Strunecka [43] reported that aluminum causes oxidative stress within brain tissue, exacerbating the clinical presentation of autism by worsening of excitotoxicity and by microglial priming. They suggested that the heterogeneous symptoms of autism spectrum disorders have a connection with dysregulation of glutamatergic neurotransmission in the brain along with enhancement of excitatory receptor function by proinflammatory immune cytokines as the underlying pathophysiological process. In this regard, dietary excitotoxins including aluminum can exacerbate the clinical presentation by worsening of excitotoxicity and by microglial priming. This opens the discussion to the use of nutritional factors that reduce excitotoxicity and brain inflammation as a maneuver to alleviate neurotoxic effects of aluminum [44].

Limitation of our study was a sample size; so a larger sample size from multiple sites is needed to improve the statistical power of this study and validate or refute its findings. Also there was sample bias for controls: the controls were chosen from the friends and neighbors of the autistic patients. This allowed an easy access to a reasonable match of geographic location and socioeconomic status but is not the most rigorous method.

*In conclusion*, there were higher levels of the heavy metals mercury, lead, and aluminum in the hair of children with autism in comparison to controls; these high levels were statistically positively correlated with some risk factors as heavy fish consumption during pregnancy, maternal smoking, and usage of anti-D and aluminum pans; however these levels were not correlated with autism severity. Biological damage from heavy metals as a neurotoxic substance beside genetic susceptibility in the form of reduced ability to excrete heavy metals and/or increased environmental exposure at key times in development may play a causal role in autism.

## Figures and Tables

**Table 1:** Statistical comparison between characteristics and risk factors of patients and controls^*∗*^.

Variables	Group				*P*
	Cases		Controls		
	*N*	%	*N*	%	
Gender					
Male	84	84.0%	74	74.0%	0.083^*∗*^
Female	16	16.0%	26	26.0%	
Age group					
<6 years	45	45.0%	44	44.0%	0.887^*∗*^
≥6 years	55	55.0%	56	56.0%	
Age					
Mean ± SD	6.24	2.43	6.80	3.04	0.152^‡^
Dental amalgam during pregnancy					
Positive	34	34.0%	15	15.0%	0.002^*∗*^
Negative	66	66.0%	85	85.0%	
House age					
>30 years	43	43.0%	1	1.0%	0.001^*∗*^
<30 years	57	57.0%	99	99.0%	
Aluminum pans					
Positive	90	90.0%	75	75.0%	0.005^*∗*^
Negative	10	10.0%	25	25.0%	
Vaccination					
Yes	99	99.0%	100	100.0%	1.00^*∗∗*^
No	1	1.0%	0	0.0%	
Rho(D) immune globulin					
Positive	25	25.0%	5	5%	0.001^*∗*^
Negative	75	75.0%	95	95%	
Fish consumption per month					
None	0	0.0%	11	15.7%	0.0001^*∗∗*^
Once	19	27.1%	19	27.1%	
2–4 times	51	72.9%	36	51.4%	
>4 times	0	0.0%	4	5.7%	
Nearby gasoline stations					
Positive	4	5.7%	2	2.9%	0.681^*∗∗*^
Negative	66	94.3%	68	97.1%	
Smoking					
Positive	32	32%	10	10%	0.0001^*∗∗*^
Negative	68	68	90	90%	

^*∗*^Chi-square test, ^*∗∗*^Fisher's exact test, and ^‡^Student's *t*-test.

**Table 2:** Comparison between studied groups as regards perinatal data.

	Group				*P*
	Cases		Controls		
	Range	Mean ± SD	Range	Mean ± SD	
Breast feeding (months)	2.0–24.0	13.5 ± 7.2	2.0–24.0	17.1 ± 4.9	0.001^*∗*^
Weaning (months)	2.0–18.0	6.1 ± 3.1	4.0–7.0	5.7 ± 0.9	0.292
Age of mother at conception (years)	17.0–40.0	26.5 ± 4.5	19.0–31.0	24.7 ± 3.0	0.005^*∗*^

^*∗*^Student's *t*-test.

**Table 3:** Comparison between cases and controls as regards heavy metal levels.

	Group						*P*
	Cases			Controls			
	Mean	±SD	Median	Mean	±SD	Median	
Lead level (mg/kg)	3.31	3.92	2.04	2.06	2.45	1.32	0.015^*∗*^
Mercury level (mg/kg)	0.39	0.37	0.28	0.25	0.16	0.20	0.023^*∗*^
Aluminium level (mg/kg)	59.19	37.98	53.00	16.78	17.31	11.11	0.0001^*∗*^

^*∗*^Mann-Whitney test.

**Table 4:** The relationship between risk factors and mercury level among cases.

Variables	Mercury level (mg/kg)		*P*
	Range	Mean ± SD	
Age group			
<6 years	0.087–1.744	0.43 ± 0.34	0.101^*∗*^
≥6 years	0.008–0.911	0.32 ± 0.22	
Dental amalgam			
Positive	0.087–0.773	0.33 ± 0.20	0.492^*∗*^
Negative	0.008–1.744	0.38 ± 0.31	
Rho(D) immune globulin			
Positive	0.117–0.149	0.13 ± 0.02	0.239^*∗*^
Negative	0.008–1.744	0.37 ± 0.28	
Fish consumption			
None	0.008–0.830	0.26 ± 0.24	**0.026** ^*∗∗*^
Once per month	0.113–0.911	0.34 ± 0.21	
2–4 times per month	0.074–0.882	0.37 ± 0.24	
>4 times per month	0.285–1.744	0.74 ± 0.68	

^*∗*^Student's *t*-test; ^*∗∗*^ANOVA, statistically significant (*P* < 0.05).

**Table 5:** The relationship between risk factors and lead level among cases.

Variables	Lead level (mg/kg)		*P*
	Range	Mean ± SD	
Age group			
<6 years	0.005–17.130	2.98** ± **4.12	0.811^*∗*^
≥6 years	0.005–21.924	3.24** ± **4.52	
Artificial feeding			
Positive	0.313–17.130	2.69** ± **3.73	0.590^*∗*^
Negative	0.005–21.924	3.31** ± **4.56	
Pica			
Positive	0.005–21.924	3.48** ± **4.92	0.507^*∗*^
Negative	0.005–16.711	2.79** ± **3.68	
Lead pipes			
Positive	0.005–21.924	3.29** ± **4.44	0.175^*∗*^
Negative	0.005–2.557	1.04** ± **0.97	
Nearby gasoline stations			
Positive	0.739–16.711	7.29** ± **7.41	**0.047** ^*∗*^
Negative	0.005–21.924	2.88** ± **4.02	
Fish consumption per month			
None	0.313–8.750	3.26** ± **2.73	0.802^*∗∗*^
Once per month	0.005–21.924	2.62** ± **4.82	
2–4 times per month	0.005–17.130	3.49** ± **4.71	
>4 times per month	0.744–4.396	2.02** ± **1.66	

^*∗*^Mann-Whitney test, ^*∗∗*^Kruskal-Wallis test.

**Table 6:** Comparison between selected risk factors and aluminum hair levels among autistic cases.

	Aluminum level (mg/kg)			*P*
	Mean	±SD	Median	
Age group				
<6 years	63.89	35.51	58.12	0.101^*∗*^
≥6 years	55.35	39.80	48.91	
Fish consumption				
Yes	49.34	19.68	58.12	0.880^*∗*^
No	59.50	38.43	52.93	
Dental amalgam				
Positive	55.21	42.73	41.14	0.205^*∗*^
Negative	61.25	35.46	54.87	
House age				
1	58.06	34.78	52.93	0.931^*∗∗*^
2	60.05	40.51	53.08	
Rho(D) immune globulin				
Positive	55.30	25.43	54.82	0.994^*∗*^
Negative	59.40	38.61	52.93	
Aluminum pans				
Positive	61.71	38.46	53.92	**0.04** ^*∗*^
Negative	36.58	24.78	30.64	

^*∗*^Student's *t*-test.

**Table 7:** Comparison between CARS degrees and levels of heavy metals.

	CARS classification						*P*
	Mild to moderate			Severe			
	Mean	SD	Median	Mean	SD	Median	
Mercury level (mg/kg)	0.31	0.3	0.25	0.43	0.41	0.34	0.132^*∗∗*^
Lead level (mg/kg)	2.27	1.98	1.93	3.87	4.56	2.3	0.171^*∗∗*^
Aluminum level (mg/kg)	60.6	36.68	58.12	58.44	38.92	49.47	0.646^*∗*^

^*∗∗*^Mann-Whitney test, ^*∗*^Student's *t*-test.
